# A realistic model for vasectomy reversal training using swine testicles

**DOI:** 10.1590/acb383023

**Published:** 2023-08-28

**Authors:** Deivid Ramos dos Santos, Wender de Jesus Pena Corrêa, Lívia Guerreiro de Barros Bentes, Rafael Silva Lemos, Victor Matheus Mendonça de Araújo, Gabrielly Leite Andrade, Renan Kleber Costa Teixeira, Luís Otávio Amaral Duarte Pinto, Herick Pampolha Huet de Bacelar

**Affiliations:** 1 Universidade Estadual do Pará – Belém (Pará) – Brazil.; 2 Universidade Federal do Pará – Belém (Pará) – Brazil.; 3 Centro Universitário do Pará – Belém (Pará) – Brazil; 4 Universidade Federal de São Paulo – São Paulo (São Paulo) – Brazil.

**Keywords:** Vasovasostomy, Microsurgery, Urologic Surgical Procedures, Low Cost Technology, Simulation Training

## Abstract

**Purpose:**

To evaluate the viability of the porcine vas deferens as a realistic microsurgical training model for vasectomy reversal

**Methods:**

The model uses swine testicles (vas deferent), which are usually discarded in large street markets since they are not part of Brazilian cuisine. The spermatic cord was carefully dissected, and the vas deferens were isolated, measuring 10 cm in length. A paper quadrilateral with 5 cm^2^ was built to delimit the surgical training field. The objective of the model is to simulate only the microsurgical step when the vas deferens are already isolated. The parameters analyzed were: feasibility for reproducing the technique, patency before and after performing the vasovasostomy, cost of the model, ease of acquisition, ease of handling, execution time, and model reproducibility.

**Results:**

The simulator presented low cost. All models made were viable with a texture similar to human, with positive patency obtained in 100% of the procedures. The internal and external diameters of the vas deferens varied between 0.2-0.4 mm and 2-3 mm, respectively, with a mean length of 9 ± 1.2 cm. The total procedure time was 43.28 ± 3.22 minutes.

**Conclusions:**

The realistic model presented proved to be viable for carrying out vasectomy reversal training, due to its low cost, easy acquisition, and easy handling, and providing similar tissue characteristics to humans.

## Introduction

Vasectomy is a safe and relatively simple male sterilization surgical procedure whose efficiency has made it one of the most popular contraceptive methods worldwide^1^,^2^. It is estimated that around 60 million men performed it^3^. However, the death of a child, early age, divorce, and new relationships are reasons to regain male fertility^4^,^5^. From this, numerous techniques were developed to recover fertility, such as percutaneous epididymal sperm aspiration and testicular sperm aspiration, both associated with in-vitro fertilization or intracytoplasmic sperm injection^6^-^8^.

However, due to its superior cost-benefit ratio compared to the other ones, vasectomy reversal has become the most preferred fertilization method for vasectomized men^5^-^8^. This procedure can be performed in two different ways: vasovasostomy or vasoepididymostomy, both technically evolving over the years to improve post-surgical results^5^,^6^. However, unlike vasectomy, its reversal is a microsurgical procedure that depends on good clinical judgment, its feasibility, and, mainly, technically qualified professionals for its execution^6^,^7^.

In addition, due to its high level of complexity, this technique becomes extremely challenging for the less experienced urologist, since it demands years of training in microsurgery, which, until recently, were performed directly on human beings, increasing the risk of damage and complications^5^. In this sense, the use of training models constitutes a crucial step in the development and improvement of microsurgical skills, before performing any procedure in humans^6^,^9^,^10^.

There are different types of microsurgical simulators for training: synthetic ones, for example, are widely used in initial learning, but they are generally expensive, do not faithfully portray tissue characteristics, and are only present in large reference centers^9^-^11^; in-vivo simulators, in turn, provide realistic characteristics to the surgical procedure, but depend on the approval of an ethics committee for the use of animals, whose tendency is to increasingly restrict the use of animals for practical training^11^.

Thus, the search for models for realistic training and high fidelity to surgical practice, without disrespecting the current ethical norms or restricting the gain of technical skills, is being sought^12^,^13^. Based on this, the present study aimed to evaluate the feasibility of a discard animal model for training in vasectomy reversal.

## Methods

It was an experimental and cross-sectional study carried out at the Laboratory of Experimental Surgery at the Universidade do Estado do Pará, following the Brazilian laws on the use and creation of animals (Law No. 11.794/08), obtaining the opinion of a waiver by the Committee of Ethics in the Use of Animals, since the product used in this study is discarded in large street markets because it is not part of the diet of the local population.

Testicles and spermatic cords were obtained after mandatory castration of pigs for meat marketing (Decree No. 9013 of March 29, 2017, Article 104) at a local breeding facility. The material was stored under refrigeration (5°C) and placed at room temperature (26°C) 1 hour before the procedure.

The performance of the microsurgical technique followed the guidelines proposed by the literature^13^-^15^ using a video system composed of a Sony Handycam HDR-XR160 high-definition (HD) camera, with 40x zoom, a 42-inch LED HD television and a digital HDMI cable, in addition to common lighting fixtures adapted to the camera providing adequate lighting for the procedure.

The present simulator only allows the execution of the anastomosis and not the complete surgery, without anesthesia, incision in planes, and hemostasis. Therefore, the creation of the training model followed the steps described ahead. First, with the aid of latex gloves, a cardboard, a stapler and common scissors, quadrilaterals measuring 5 cm × 5 cm were made with two holes on opposite sides, one for entry and one for exit^10^,^16^. Then, the testis and the spermatic cord were washed with running water and soap to remove impurities.

Subsequently, the funiculus was completely dissected, from its origin to the proximal stump, where it ruptured during castration of the pig, and the vas deferens were isolated in the quadrilateral. After that, a total cross-section of the canal was performed with the aid of curved Metzenbaum scissors.

**Figure 1:**
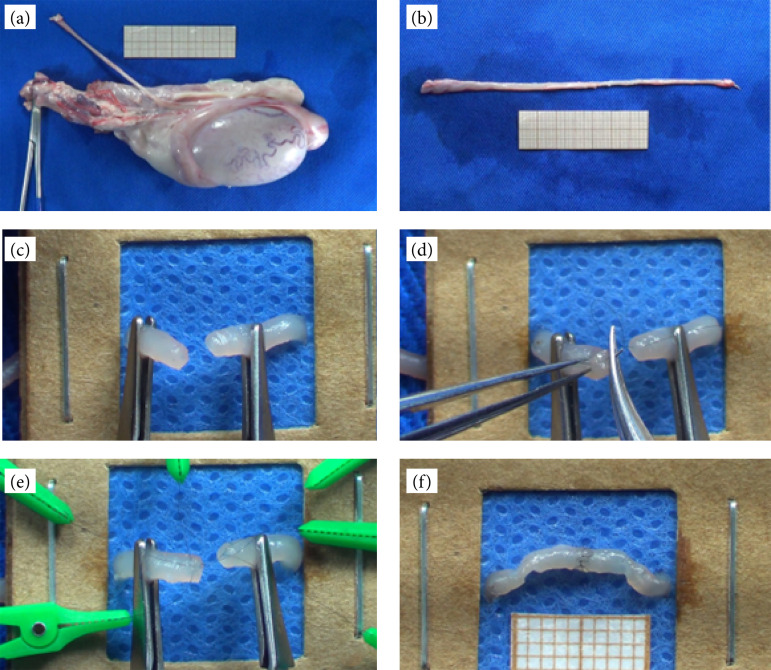
Microsurgical steps of the training model. **(a)** Swine testicle after washing and thawing. **(b)** Duct deferens dissected and removed from the testis. **(c)** Deferent duct transversely sectioned. **(d)** Performing the first transfixation in the duct with 10-0 nylon thread. **(e)** Deferent duct transfixed with 10-0 nylon threads. **(f)** Completely sutured vas deferens.

Source: elaborated by the authors.

**Figure 2:**
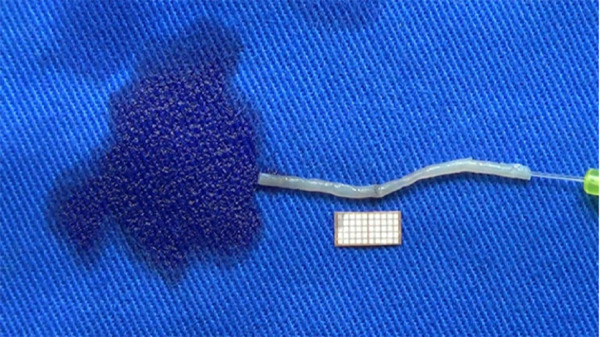
Sutured vas deferens submitted to the patency test.

Source: elaborated by the authors.

The reversal technique chosen was vasovasostomy, characterized by eight equidistant simple stitches (four at 3, 6, 9 and 12 o’clock and four interspersed), made with 10-0 nylon thread, coapting the entire circumference, without affecting the lumen^17^. The instruments used to perform the technique were: watchmaker’s tweezers, a needle holder and curved microsurgical scissors. Patency was tested with the introduction of a 24G Jelco, with a slow injection of 5 to 10 mL of 0.9% saline solution (SF 0.9%), or a 2-0 Prolene thread, replacing the Jelco, when the injection liquid did not flow properly Figs. 1 and 2).

In all, five models were created, and all procedures were performed by only one researcher, with more than 10 years of experience in the area, to avoid bias in the research. The parameters analyzed were: feasibility for reproducing the technique, patency before and after performing the vasovasostomy, cost of the model, ease of acquisition, ease of handling, execution time and model reproducibility. Microsoft Word 2016 and Excel 2016 software were used for data analysis and image editing.

## Results

All models made proved to be viable for carrying out vasectomy reversal training, with positive patency obtained in 100% of them. The materials used were easy to acquire and had no cost. The human-like consistency of the vas deferens provided adequate handling during the procedure.

The luminal diameter and external diameter of the vas deferens varied between 0.2-0.4 and 2-3 mm, respectively, with a mean length of 9 ± 1.2 cm. It was possible to perform the technique up to three times on each model. Vas deferens shorter than 6 cm were discarded, since they tend to disconnect from the quadrilateral exit orifice, making the technique difficult.

The mean total procedure time was 43.28 ± 3.22 minutes. Local humidification with jets of 0.5 mL of 0.9% SF, with the aid of a 10-mL syringe, was necessary during the execution of the technique. This resulted in an increase of 2.86 ± 0.24 minutes in the total procedure time, given that the mean interval for humidification was 3.00 ± 0.46 minutes.

## Discussion

Vasectomy reversal is a microsurgical procedure that consists of the anastomosis of the vas deferens–vasovasostomy–or the vas deferens and the epididymis–vasoepididymostomy–, to restore fertility as a result of a previous vasectomy^5^,^6^,^8^.

Despite their high efficiency, most high-fidelity simulators require high-cost technologies for their manufacture, such as three-dimensional models^6^,^10^,^18^. This restricts the use of these technologies to large training centers. On the other hand, the present model did not present costs, since the components–swine testicle and spermatic cord–are commonly discarded by the breeding sites, allowing the training to be carried out anywhere.

The five models made allowed their reuse up to three times each, with 15 procedures being performed in total, obtaining positive patency in 100% of the attempts. There was impairment in the quality of the procedure with a greater number of attempts, due to tissue wear with frequent handling. This fact suggests the considerable evolution of skills that the model can offer, due to the number of repetitions provided by it, although they are limited^5^,^6^,^9^,^10^,^12^,^15^,^19^. The average time of each procedure (43.28 ± 3.22 minutes) was similar to that one found^10^, with 6.6% of this time being allocated to humidifying the duct due to high adherence to latex due to dehydration, and, although time is an important evaluation variable, the quality of the technique is paramount^19^.

According to Patel and Smith^5^, the human vas deferens has a luminal diameter that can vary from 0.3 to 0.4 mm and the epididymal from 0.15 to 0.2 mm. Vasoepididymostomy technique is more challenging than vasovasostomy for most surgeons, making the latter the most performed. This makes clear why the latter technique was used in the model, as the most complex one must be performed after a considerable level of learning^20^. In addition, the vas deferens presented tissue consistency similar to that of humans, and the luminal duct had a variable diameter of 0.2-0.4 mm, increasing the fidelity of the model to reality.

It is worth noticing that the present study had limitations. Avulsion of the spermatic cord during castration^18^ can reduce the internal diameter of the vas deferens, making it impossible to perform the patency test, in addition to reducing the length of the vessel to an unviable size for use in the model (< 6 cm). In addition, the use of video magnification systems, despite reducing the risks of contamination, as well as the costs compared to a conventional microscope, limits the initial stages of training because they do not present three dimensions, which can be circumvented after a previous period of adaptation^13^,^20^. Even so, the model offers high fidelity to urological practice, becoming an ally in the acquisition of microsurgical skills.

## Conclusion

It was concluded that the porcine vas deferens proved to be viable for carrying out vasectomy reversal training, since the dimensions of its walls and consistency are similar to human ones, obtaining positive patency in the five models tested. In addition, the easy acquisition, low cost, absence of special conditions for use, respect for ethical principles of reducing the unnecessary use of animals, and reproducibility in several training centers make this model attractive.

## Data Availability

The data will be available upon request.
